# Out-of-Plane Auxetic Behavior in Cellulose Nanofibril Films

**DOI:** 10.1021/acsomega.4c09915

**Published:** 2025-03-26

**Authors:** Fariha Rubaiya, Meisha L. Shofner, Lauren M. Garten

**Affiliations:** 1 School of Materials Science and Engineering, Georgia Institute of Technology, Atlanta, Georgia 30332, United States; 2 Renewable Bioproducts Institute, Georgia Institute of Technology, Atlanta, Georgia 30332, United States

## Abstract

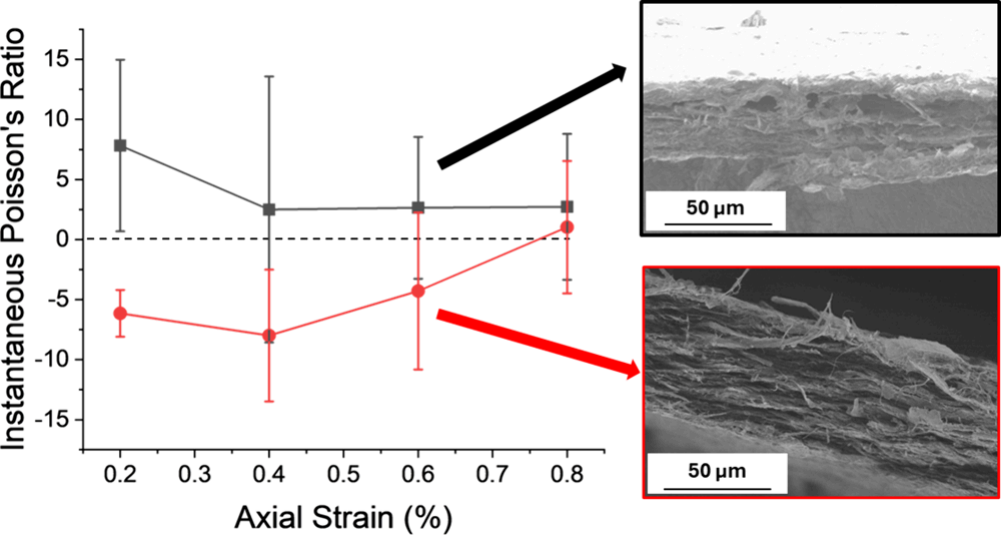

While auxeticity has been established in cellulose-based paper and paperboards and computational studies suggest auxetic behavior should occur within cellulose structures, the auxetic response of neat cellulose nanofibril (CNF) films has not yet been experimentally established. Here, we show that an out-of-plane auxetic response does occur in CNF films and that the magnitude of the response is dependent on the film density, microstructure, and sample geometry. CNF films are fabricated using vacuum filtration, followed by conditioning at 23 °C and 50% relative humidity. The CNF aqueous suspension amount is varied from 90 to 450 mL, which progressively increases the film density until reaching a plateau of 1.05 ± 0.02 g/cm^3^ for films with suspension volumes equal to or greater than 270 mL. Aside from varying the film densities, the aspect ratio of the film is varied from 1.5 to 5 (ratio of rectangular CNF film length to width) to determine how the sample dimensions contribute to the auxetic response, specifically if stress fields associated with gripping of the sample constrain the fiber network. From these studies, CNF films with a density of 1.05 g/cm^3^ and a film aspect ratio of 5 exhibit the highest auxetic response in the elastic region with a negative Poisson’s ratio of −5.3 as determined by linear fitting and the largest instantaneous Poisson’ ratio value of −7.99 at 0.4% strain. Overall, this work provides insight into the processing–structure–property relationships that define auxeticity in CNF films, which can enable the use of CNFs as auxetic metamaterials in a broad range of applications such as sensing, protective gears, composites, and structural materials.

## 1 Introduction

While most materials exhibit a positive Poisson’s ratio, auxetic materials have a negative Poisson’s ratio where expansion occurs not only in the direction of applied force but in at least one secondary perpendicular direction.^1−3^ Auxetic behavior, also termed auxeticity, provides advantageous mechanical behavior including improved fracture toughness,^4−6^ resistance to indentation,^7−9^ shear rigidity,^10−12^ and enhanced vibrational damping.^13,14^ Due to the unique properties associated with auxeticity, significant research has been dedicated to understanding the underlying deformation mechanism behind it.^15,16^ Auxeticity has previously been observed in materials that contain cellulose, but the magnitude of the auxetic response and the underlying mechanisms that result in a negative Poisson’s ratio varies significantly depending on the materials’ microstructure and/or metamaterial structure.^17−19^ Although cellulose is the most abundant organic polymer on earth, the presence and driving mechanisms behind auxetic behavior in nanocellulose have not yet been established.

Cellulose is a linear polymer, containing two anhydro-d-glucopyranose (AGU) rings joined by a β-1,4-glycosidic bond. The cell wall of wood is a complex hierarchical structure consisting of cellulose microfibrils as the primary load bearing units. Several studies have reported possible auxeticity in cellulose and its structures. In 1948, Hearmon proposed the existence of a negative Poisson’s ratio in wood in multiple directions using elastic tensors for a hardwood and softwood in the transverse–longitudinal (TL) plane.^20^ Sliker and Yu found strains both parallel and perpendicular to the loading direction to be positive in cottonwood, brasswood, and soft maple, indicating an auxetic response.^21^ A meta-study conducted by Marmier et al. showed that auxeticity in wood is so prevalent that it occurred in 87 out of 123 of their chosen hardwood samples and 58 out of 62 softwood samples.^22^ According to their results, a negative Poisson’s ratio would be expected in woods that have a density lower than 0.8 g/cm^3^ where stress is applied along specific angles that slightly deviate from the longitudinal direction (off-axis).

In addition to wood, auxeticity has been experimentally observed and computationally evaluated in ramie and kraft fibers, which mainly consist of crystalline cellulose. A negative Poisson’s ratio has been reported through X-ray diffraction in commercially bleached ramie fibers treated with NaOH.^23^ The crystal structure of cellulose in these fibers was cellulose II, which is one of several polymorphs for cellulose.^24^ The mechanisms producing auxeticity were attributed to the rotation of the cellulose chains perpendicular to the uniaxial stress.^25^ Auxeticity has also been observed in another cellulose polymorph, cellulose I_β_ (kraft cooked Norway spruce), by monitoring the reflections of the (200) and (004) planes via X-ray diffraction before and after stretching to the yield point.^26^ Yao et al. identified two potential mechanisms of this behavior through molecular modeling of 2D projections of the cellulose I_β_ structure, namely, rotation and/or unfolding of the cellobiose chain along the loading direction.^27^

Auxeticity has also been observed in structures composed of cellulose fibers, such as paper.^17,28,29^ Paper is made by breaking down wood fibers through a pulping process, followed by forming and drying the cellulose fibers into sheets.^30^ Just as in raw wood, a negative Poisson’s ratio has been reported in a variety of papers. A modeling study by Cox indicated that paper is expected to exhibit a negative Poisson’s ratio in-plane but demonstrates a conventional (positive) Poisson’s ratio in the out-of-plane direction.^31^ However, Öhrn and Stenberg and Fellers tested the out-of-plane Poisson’s ratios of several kinds of paper and found them to be negative.^28,29^ The out-of-plane auxetic behavior of commercially available papers such as copy paper, paperboard, filter paper, and cotton paper have been tested by measuring thickness changes due to tensile loading.^17^ While all of these papers exhibited an auxetic response, copy paper showed the largest negative Poisson’s ratio of −3. Variations in Poisson’s ratio were attributed to the processing conditions and their impact on fiber orientation and the fiber–fiber interactions within the fiber network structure. The presence of strong hydrogen bonds at cellulose fiber intersections is thought to be a significant factor in their unique auxetic response.^17^ In addition to paper, auxeticity has also been studied in bacterial cellulose and microfibrillated cellulose films using Raman spectroscopy, which revealed an in-plane auxetic response of the fibrous networks.^32^ The mechanism of this auxetic behavior has been attributed to the reentrant nature of the fiber network or potentially due to the transverse strain occurring perpendicular to the loading direction because of the strong bonding within the random arrangements of the fibers. These studies indicate that the auxetic response, which is prevalent in fiber networks, depends on the reorientation and interactions of fibers under strain, whereas the auxetic response in the molecular or nanoscale in cellulose structures depends on the deformation of cellobiose chains.

While conventional paper is made from cellulose fibers with dimensions on the millimeter scale, smaller-scale cellulose fibers can be produced through further deconstruction of the wood cell wall.^33^ Such nanoscale cellulose fibers are termed nanocellulose or cellulose nanomaterials (CNMs). CNMs are available in several types, one of which is cellulose nanofibrils (CNFs). CNFs are defined as having an aspect ratio greater than 10 (10–100 nm in diameter and several μm in length) with both ordered and disordered regions.^34,35^ The disordered region provides flexibility to the material.^24^ CNFs are reported to have a Young’s modulus of approximately 138 GPa and a tensile strength of 2–3 GPa.^36,37^ Like larger scale cellulose fibers, CNFs may be made into films or papers from an aqueous suspension through a method that removes the liquid component to allow physical interactions between the fibrils and mechanical entanglement. The strength of these films relies on the fibril-to-fibril interaction facilitated by hydrogen bonding between the cellulose molecules. The strong bonding between the fibrils enables uniform stress distribution during deformation, enhancing the tensile strength and toughness of the CNF films.^34,38^ The mechanical properties of CNF-based papers is significantly altered by controlling the CNF dimensions, degree of polymerization (DP), porosity, moisture content, fiber alignment, and surface functionality.^38^ CNF-based papers and composites have also garnered attention in a variety of electrical and biomedical applications because of their tunable mechanical properties.^39,40^

While the mechanical properties of CNF films and composites have been explored widely, auxeticity in CNF films has not yet been established. Given the auxetic response of conventional paper and other fiber network materials, it is reasonable to expect that CNF films would also show an auxetic response since these films more closely mimic the structure of paper than films made from shorter CNMs such as cellulose nanocrystals. Additionally, the expected fiber–fiber interactions (interfiber physical bonding and fiber entanglement) would be similar to those in paper. Auxeticity in CNF films, as well as the potential to modulate the response through processing parameters, would enable expanded applications for CNFs in areas such as substrates for sensing, energy harvesting, and bioelectronics as well as filtration and encapsulation. Therefore, the goal of this work is to discover the attributes of CNF networks that enable auxeticity in order to provide guidelines for producing auxetic metamaterial CNF structures.

In this study, we demonstrate the impact of processing on the presence and magnitude of the auxetic behavior in neat CNF films. A comprehensive investigation into the auxetic behavior of CNF films fabricated through vacuum filtration with varying density, thickness, and sample dimensions (i.e., aspect ratio defined as the ratio of sample length to width) is conducted. Our findings show that varying density influences the internal film structure and the thickness affects the porosity distribution in the films. Beyond the microstructure of the fiber network, external constraint affects the auxetic response, with sample gripping imposing a stress field that hinders the auxetic response. This condition would affect device designs by using these films. Ultimately, these results provide a foundational framework for tailoring auxetic behavior in nanocellulose films, which is needed to capitalize on this response and to use them as mechanical metamaterials in functional applications.

## 2 Experimental Section

### 2.1 Materials

A 3 wt % aqueous suspension of CNFs (CAS No: 9004-34-6) was purchased from the Process Development Center of the University of Maine. Lab-grade deionized (DI) water was used for film fabrication. All of the items were used without any further modification.

### 2.2 CNF Film Fabrication

A 3 wt % aqueous suspension of CNFs was used as the precursor material for CNF films. According to the manufacturer, the nanofibrils range in length from a few hundred micrometers to millimeters. The average diameter of the CNFs was assessed by atomic force microscopy (AFM, Dimension Icon, Bruker). To evaluate CNF dimensions, the 3 wt % suspension was diluted to 0.001 wt % by adding DI water, followed by casting onto a silicon wafer and then drying at room temperature overnight. AFM data were acquired in tapping mode, and CNF diameter was determined by measuring the height of individual fibrils. A total of 50 measurements were taken to ensure reliability.

**Figure 1:**
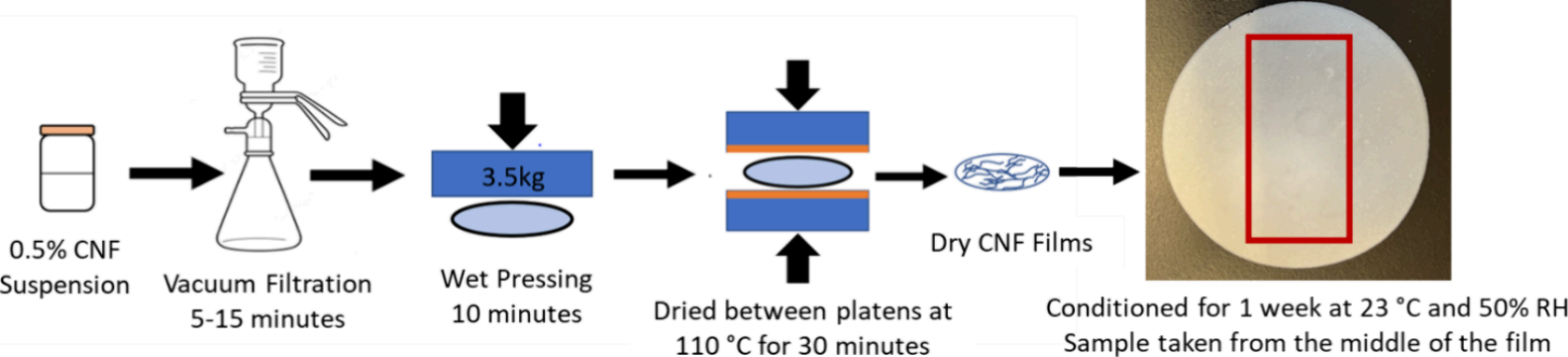
Schematic representation of CNF film fabrication.

Films were fabricated from the CNF suspension using a vacuum filtration method similar to that described in a previous report.^41^Figure 1 shows the CNF thin film fabrication process schematically. As a first step, the CNF 3 wt % aqueous suspensions were diluted to 0.5 wt % by adding DI water. DI water and the CNFs were mixed at 3200 rpm in a vortex mixer (VWR Cat No 76549–928) to produce a homogeneous suspension before vacuum filtration was undertaken. The solid loading in the suspension was kept at 0.5 wt % to ensure a uniform and stable suspension. The thickness and density of films produced from the CNF suspension were varied by changing the amount of suspension used (90, 180, 270, 360, or 450 mL). In this work, the films are referred to by the amount of suspension that was used, i.e., the CNF-90 film was made using 90 mL of the CNF suspension. CNF films were fabricated from the prepared CNF suspensions using vacuum filtration through Whatman filter paper (Cat No 1001–150, pore size 11 μm) using a mini diaphragm vacuum pump (LABOPORT UN 86KT.45p, vacuum pressure: 160 mbar) for filtration and dewatering. The resulting films were circular, measuring approximately 13 cm in diameter. After film formation, the films were wet pressed under a 3.5 kg weight for 10 min to remove excess water. Then, the wet films were subjected to additional drying by putting the films between heated platens in a hydraulic press (Carver model 4386, 12-ton capacity) at 110 °C for 30 min. This step was performed to ensure that the films remained flat. No additional pressure was applied during the drying process. Following this heating step, the dry films were conditioned at 23 °C and 50% relative humidity (RH) for a week before any mechanical testing was undertaken according to the TAPPI T 402 standard.^42^

### 2.3 Sample Preparation and Characterization

Rectangular samples were cut from the middle of the conditioned CNF films for testing (indicated by the red rectangle in Figure 1) as the thickness in this region was more uniform compared with the edges. To investigate the effect of different sample dimensions/strain rates/external constraint on the auxetic response, the CNF-270 films were cut into rectangular samples with three different lengths while maintaining a constant width of 1.8 cm. The aspect ratio (AR) is defined as the ratio of the length of the rectangular CNF film to its width. The aspect ratios used were AR 1.5 (gauge length 2.7 cm), AR 3 (gauge length 5.4 cm), and AR 5 (gauge length 9 cm).

Film density was calculated by dividing the mass of each cut sample by its volume. The mass was measured, post conditioning treatment, in an analytical balance (Fisher Science Education). The thickness of the samples was measured using a digital micrometer (Mitutoyo Disc Micrometer-Non-Rotating Spindle, Series 369). The flat disks in the micrometer were 2 cm in diameter and exerted a 7 N force, while the thickness was recorded. The wide contact area of the flat disks compensated for the surface roughness of the CNF films, and the constant force applied reduced the effect of local thickness variations on the thickness measurements, allowing for more consistent measurements. The average thickness was calculated from three to five measurements taken at different positions along the length of each sample. Also, this measurement was carried out on at least five samples of each category. By taking multiple measurements across several samples, it was ensured that any variability due to local surface roughness was averaged out, providing reproducible data. Porosity was then calculated using the difference between the theoretical density of CNFs and the measured density of the CNF film, as shown in eq 1.^43^ The theoretical value of density used for CNF was 1.5 g/cm^3^.^44^ Grammage was also calculated by dividing the mass of the film by square meter of area.
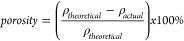
1

The surface morphology, internal structure, and porosity of the samples were qualitatively assessed by using scanning electron microscopy (SEM, Hitachi SU8010). The samples were sputter-coated with gold–palladium and imaged by using an accelerating voltage of 1–3 kV. Thermogravimetric analysis (TGA, TA Instruments Q50) was conducted to determine the moisture content of the conditioned CNF films. For TGA analysis, the samples were heated at a heating rate of 10 °C/min from room temperature to 800 °C, with an isothermal step at 100 °C for 10 min. The weight change before and after the isothermal step was used to calculate the moisture content of the films.

### 2.4 Mechanical Testing

To measure the elastic modulus and tensile strength, tensile tests were conducted using a universal mechanical testing frame with a 1 kN static load cell and microtensile grips (Instron model 68TM-R5566). The samples were extended at a constant rate of 0.5 mm/min. The elastic modulus was calculated using the slope from the initial linear region (0.15–0.4% strain) of the stress–strain curve, and tensile strength was derived from the point of maximum stress before tensile failure. Statistical tests, one-way ANOVA followed by post hoc Tukey tests, were conducted to assess the statistical difference among the sets of data.

To determine the auxetic response of the films, the thickness of CNF films was measured in the middle of the sample gauge length during tensile testing at specific intervals. To allow time for the thickness measurements, the tensile test was paused at intervals of 0.2% strain within the elastic deformation region and at 1% strain intervals in the plastic deformation region until failure. Each interval lasted for 30 s, giving enough time to measure the thickness of the sample again using the same digital micrometer used to make the initial thickness measurements described above. The films were marked with a circular area corresponding to the micrometer plate diameter to ensure that the thickness was measured in the same area for each subsequent measurement. The value of Poisson’s ratio was calculated using the thickness strain and the axial strain. An increase in sample thickness with increasing extension indicated a negative out-of-plane Poisson’s ratio and thus auxetic behavior.

For all of the mechanical tests, five samples were tested for each sample type. All of the mechanical tests were performed at ambient humidity and temperature. The tests were performed shortly after removing the films from the conditioning chamber, held at 23 °C and 50% RH, to ensure that the samples had similar atmospheric water exposure prior to testing. This testing process was repeated for films from different starting suspensions, as well as films with varied aspect ratios.

### 2.5 Calculating Auxeticity

To evaluate the auxeticity of CNF films, the Poisson’s ratio was calculated. The Poisson’s ratio can be determined through several approaches; each approach offers distinct insights into materials deformation behavior. Values for the out-of-plane Poisson’s ratio were calculated using engineering strain in eq 2.
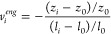
2

Here, *l*_i_ and *z*_i_ indicate the sample length and thickness value, respectively, at a given strain level, while *l*_0_ and *z*_0_ denote the initial length and thickness of the sample. This value is referred to as the “effective” Poisson’s ratio or engineering Poisson’s ratio. This gives information regarding how the material deforms with respect to its initial condition. The Poisson’s ratio was also calculated using linear fitting over the elastic strain values. By analyzing the slope of the thickness strain vs axial strain, the linear Poisson’s ratio could be calculated for the elastic region.

Additionally, values of the instantaneous out-of-plane Poisson’s ratio (ν_i_) were calculated from eq 3. The instantaneous true strain was used from measurements taken at every *i*-th hold position as follows:
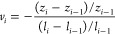
3

Here, *l*_*i*_ refers to the sample length at a given strain value, and *z*_*i*_ denotes the thickness value at the same strain value. *l*_*i*–1_ and *z*_*i*–1_ represent the corresponding values of length and thickness at the preceding strain level (*i* – 1). When the deformation behavior is not linear, the engineering Poisson’s ratio does not properly represent how a material deforms under instantaneous or sudden loading condition, but rather those relative to the initial conditions.^45^ Thus, to understand the CNF progression of deformation behavior in small strains, the instantaneous Poisson’s ratio was analyzed.

### 2.6 Swelling Test

Swelling tests were performed using 20 mm × 18 mm rectangle samples cut from the different sets of conditioned CNF films. Samples were prepared and weighed at ambient conditions and then submerged in DI water for 1, 12, 24, and 48 h. Because the weight plateaued after 1 h of being submerged in water for all samples, the data obtained after 12 h of water exposure was used for evaluation. After 12 h, the films were taken out of the water and blotted on weighing paper twice on both sides to remove excess surface water. Then, the weight of the films was measured after blotting. This test is a modified version of a previous study where the weight change due to water absorption was calculated as the ratio of the weight difference before and after water submersion to the initial dry weight.^46^ The water uptake of the films provided another perspective into their porosity, as higher water absorption indicated a more open and porous structure. Since porosity and fiber–fiber interaction are inversely related,^44^ films with lower swelling behavior suggested a denser structure with higher fiber–fiber interaction.

## 3 Results and Discussion

The type of CNF used to make the films is held constant in this work. These CNFs have an average diameter of 21.3 ± 6.5 nm, as measured by AFM. However, the amount of material used to make the films is varied. The thickness, density, porosity, and grammage of the films produced with various suspension amounts and aspect ratios are given in Table 1. The thickness of the films increases as the amount of suspension increases. The density and porosity of the films also change with changing suspension amounts. Thicker films have higher densities and lower porosities, suggesting changes in the film structure. This trend reaches a plateau at a thickness of ∼100 μm. Above this thickness, the films have similar densities and porosities. The grammage values also increase as the amount of CNF suspension used to produce the film increases, as expected. TGA results for the conditioned CNF films show that the moisture content is constant at 4–5 wt % (Figure S1) in all films and is not dependent on film thickness or density.

**Table 1:** Thickness, Density, Porosity, and Grammage Values for CNF Thin Films Processed from Varying CNF Suspensions

CNF films	film aspect ratio (AR)	CNF aqueous suspension volume (mL)	thickness (μm)	density (g/cm^3^)	porosity (%)	grammage (g/m^2^)
CNF-90	5	90	44 ± 3	0.76 ± 0.03	49 ± 2	34 ± 1
CNF-180	5	180	76 ± 5	0.94 ± 0.02	37 ± 1	72 ± 4
CNF-270	1.5	270	99 ± 4	1.04 ± 0.03	30 ± 2	103 ± 5
	3	270	98 ± 3	1.05 ± 0.04	29 ± 3	103 ± 5
	5	270	98 ± 4	1.05 ± 0.03	30 ± 3	102 ± 5
CNF-360	5	360	130 ± 8	1.04 ± 0.03	31 ± 2	135 ± 8
CNF-450	5	450	162 ± 3	1.07 ± 0.02	28 ± 2	175 ± 5

### 3.1 Impact of the Film Aspect Ratio on the Auxetic Response

**Figure 2:**
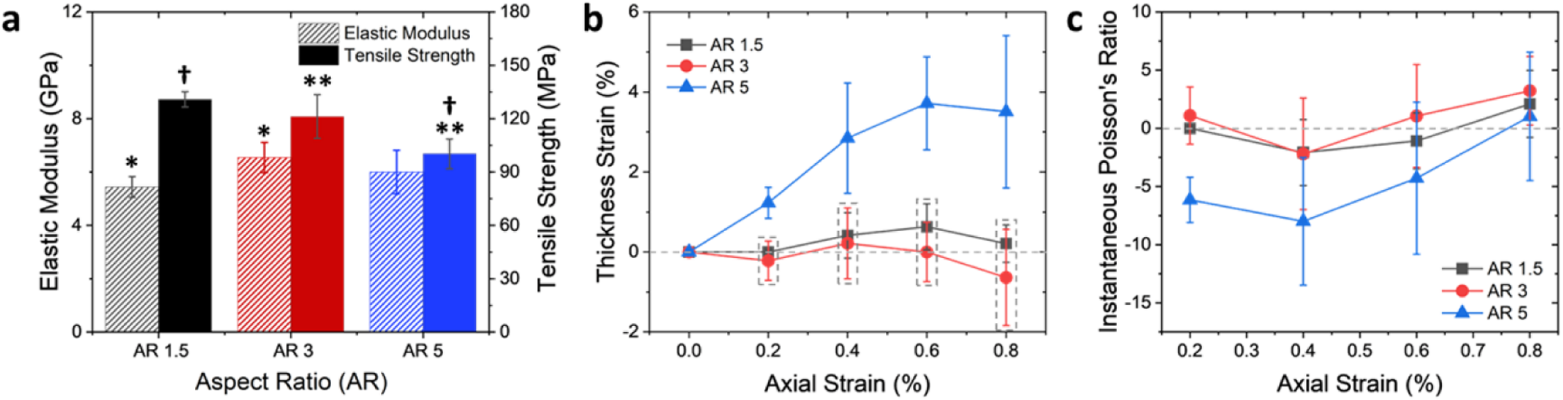
Properties from tensile testing of the CNF-270 films with different aspect ratios: (a) elastic modulus and tensile strength, (b) thickness strain, and (c) instantaneous Poisson’s ratio as a function of elastic axial strain. For the elastic modulus and tensile strength data, values marked with the same symbols are statistically different from each other. For the strain data, values inside dashed rectangles are not statistically different from each other.

Initial tensile testing reveals that the upper limit of the elastic region of the CNF films lies within 0.6–0.8% strain. Beyond this limit, the films begin to plastically deform. Representative stress–strain curves for films with varying aspect ratios are presented in Figure S2. The continuous stress–strain curves indicate that the films with different geometries exhibit similar behavior in the elastic region but fail at different tensile strengths in the plastic region. Figure S3 shows the stress–strain curve of the general tensile test overlaid with the modified auxetic-tensile test with thickness measurement intervals, showing that while stress relaxation does occur when deformation is paused, it is quickly recovered on reinitiating the test. Figure 2a shows the mechanical properties of CNF-270 films with aspect ratios of 1.5, 3, and 5. The elastic modulus of CNF-270 films with various aspect ratios ranges from 5.1 to 7.7 GPa. The average tensile strengths of the AR 1.5, AR 3, and AR 5 samples are 131, 121, and 100 MPa, respectively. One-way ANOVA followed by post hoc Tukey tests show that tensile strengths of AR 1.5 and AR 3 samples are not statistically distinct. However, the tensile strength decreases as the aspect ratio increases to 5. Samples with lower aspect ratios exhibited higher tensile strength due to the concentrated grip force during the tensile test, while in longer samples, the grip concentration was not affecting the middle of the sample. Thus, higher-aspect-ratio samples provide a more accurate representation of the material’s properties rather than the testing geometry.

The aspect ratio of the samples has a significant impact on the auxetic response (determined from changes in the thickness of each sample measured at defined axial strain intervals). Figure 2b presents a comparison of the thickness changes with increasing axial strain for the CNF-270 films with different aspect ratios. The data shown in Figure 2b are restricted to axial strains associated with elastic deformation. The AR 5 samples show an increase in thickness with increasing axial strain, thus exhibiting an auxetic response. Alternatively, the AR 1.5 and AR 3 films show smaller changes in thickness, leading to a lesser auxetic response at 0.4% and 0.6% strain. At strains that induce plastic deformation, the thickness changes for all samples become smaller, and in AR 1.5 and AR 3 samples, the thickness begins to decrease (Figure S4a). These results suggest that the stress field induced by sample gripping plays a significant role in the thickness change for samples with shorter aspect ratios, constraining the auxetic response.

To confirm that the reduced auxetic response was due to sample gripping, the influence of strain rates on the auxetic response is also considered. Although the extension rate for the tensile test is kept constant (0.5 mm/min), the strain rate decreases as the aspect ratio increases since the sample length used to calculate strain is also increasing. For AR 1.5, 3, and 5, the strain rates are 3.08 × 10^–4^, 1.5 × 10^–4^, and 9.25 × 10^–5^ s^–1^, respectively. Given these different rates, perhaps the slower strain rate is what produces an increased auxetic response, as a slower strain rate could allow the fibers more time to rearrange during deformation. To evaluate the possible effect of strain rate on auxeticity, CNF-270 AR 5 films were also subjected to a modified tensile test at an extension rate of 1.66 mm/min, which would be a strain rate of 3.08 × 10^–4^ s^–1^. The thickness strain (%) versus axial strain data for these films at strain rates of 3.08 × 10^–4^ and 9.25 × 10^–5^ s^–1^ are given in Figure S5. These data reveal that different strain rates do not significantly vary the thickness strain in the elastic region of these films, and thus, sample geometry (AR) and the associated gripping effects are the primary sources of differences seen in the auxetic response of CNF-270 films.

Figure 2c shows the instantaneous Poisson’s ratios for the CNF-270 samples for various aspect ratios. Table 2 shows the calculated values for the linearly fitted, instantaneous, and effective Poisson’s ratios for the same samples. The values from linear fitting are obtained as the slope of a linear regression fit of the data points between 0 and 0.8% strain. The other values are calculated for specific strains using the data at the strain value of interest and the data at the strain value before it as the reference point (the instantaneous Poisson’s ratio) or using the data at the strain value of interest and the data at 0% strain as the reference point (the effective Poisson’s ratio). The instantaneous Poisson’s ratios of the AR 1.5 and AR 3 samples show negative values at 0.4% strain, whereas at 0.2% and 0.8% axial strain levels, they are positive for both. The AR 5 samples start with a negative instantaneous Poisson’s ratio of −6.15 at 0.2% strain. Then, the magnitude of the auxetic response increases, and the instantaneous Poisson’s ratio becomes −7.99 at 0.4% strain. Finally, the Poisson’s ratio reaches −4.29 at 0.6% strain.

**Table 2:** Values of Poisson’s Ratios for CNF-270 Films in Varying Aspect Ratios Calculated in the Elastic Region

		instantaneous Poisson’s ratio				effective Poisson’s ratio			
CNF films	linear fitting	at 0.2% strain	at 0.4% strain	at 0.6% strain	at 0.8% strain	at 0.2% strain	at 0.4% strain	at 0.6% strain	at 0.8% strain
AR 1.5	–0.59	0.00 ± 0.00	–2.08 ± 2.84	–1.08 ± 2.41	2.10 ± 2.87	0.00 ± 0.00	–1.04 ± 1.42	–1.05 ± 0.96	–0.26 ± 0.58
AR 3	0.39	1.10 ± 2.45	–2.19 ± 4.78	1.06 ± 4.42	3.22 ± 2.9	1.1 ± 2.46	–0.54 ± 2.21	0 ± 1.24	0.8 ± 1.51
AR 5	–5.3	–6.15 ± 1.94	–7.99 ± 5.49	–4.29 ± 6.54	1.03 ± 5.51	–6.15 ± 1.94	–7.12 ± 3.4	–6.19 ± 1.93	–4.38 ± 2.38

The values of the effective Poisson’s ratio of the AR 1.5 and AR 3 samples do not follow the same trend as the AR 5 sample in the elastic region. The AR 1.5 samples show negative values of effective Poisson’s ratio from 0.4 to 0.8% strain, whereas the AR 3 samples only show a negative effective Poisson’s ratio at 0.4% strain. On the other hand, the AR 5 samples display a negative effective Poisson’s ratio throughout the elastic strain region. A visual representation showing the change in effective Poisson’s ratio with respect to axial strain in CNF films with varying aspect ratios is shown in Figure S6a. Figure S6b displays that AR 5 samples also have the most negative value of Poisson’s ratio when calculated using linear fitting in the elastic region (*v* = −5.3). This auxetic response is comparable to that found in needle punched nonwoven networks made of polyester fibers where the linear fit out-of-plane Poisson’s ratio was found to be −5.7.^47^

The effective Poisson’s ratio only depicts materials deformation relative to its initial condition. The instantaneous Poisson’s ratio, calculated from the difference in thickness strain between successive axial strain measurements, more accurately captures the progression of auxetic response for smaller deformations.^45^ This description of the auxetic response is particularly important for materials with nonlinear deformation behavior and dynamic loading conditions. The instantaneous Poisson’s ratio values of AR 5 reach positive value at around 0.8% strain and remain near zero until tensile failure (Figure S4b and Table S1). This trend indicates that a sufficient number of fiber–fiber junctions are preserved at higher axial strains, such that the thickness values remain the same. This trend also suggests that attributes of the fiber network that produce auxeticity are not the dominant pathways for deformation in the plastic strain regime. Overall, our results indicate that sample dimensions are an important parameter in terms of demonstrating auxeticity in cellulose films and would affect the device design in later work. In the lower AR samples, the fiber network is artificially constrained due to the low gauge length and resulting effect of gripping along the sample length. In contrast, AR 5 samples are not as affected by gripping since they are longer, and we are able to measure the response of the as-produced fiber network, which exhibits a more pronounced auxetic response.

### 3.2 Auxetic Response as a Function of Film Thickness and Density

**Figure 3:**
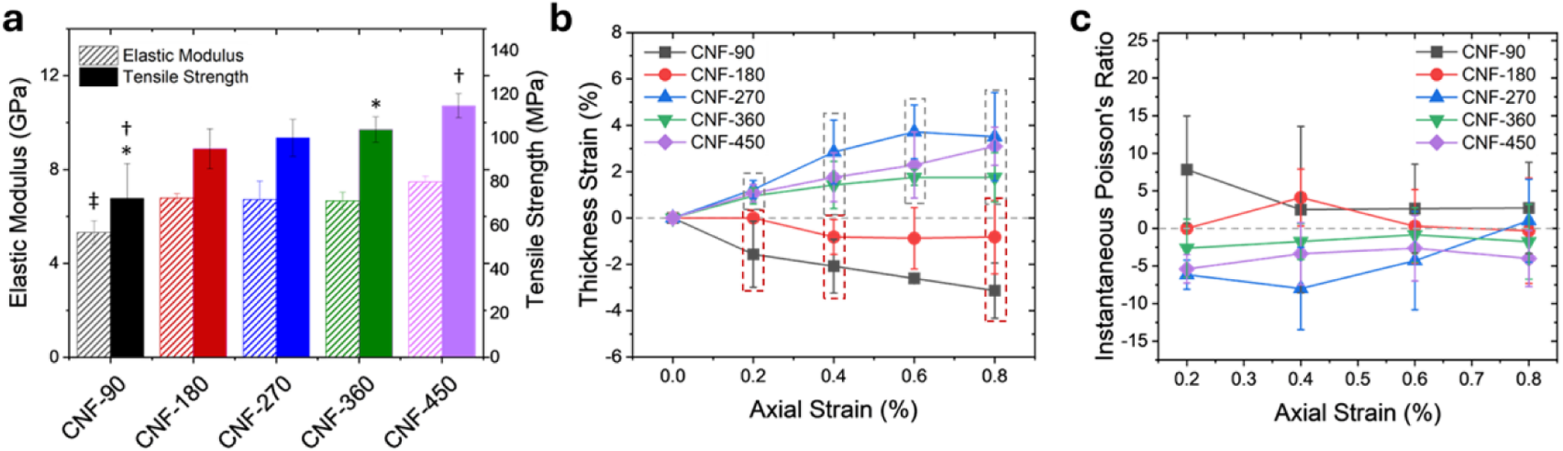
Properties from tensile testing of the CNF films made with different amounts of suspension: (a) elastic modulus and tensile strength, (b) thickness strain as a function of axial strain in the elastic region, and (c) instantaneous Poisson’s ratio values for elastic deformation. For the tensile strength data, values marked with the same symbols are statistically different from each other, and the elastic modulus value marked with ‡ is statistically different from the other modulus values. For the strain data, values inside of dashed rectangles are not statistically different from each other.

The thickness and density of CNF films can greatly alter their structural integrity, transparency, thermomechanical, and mechanical properties,^48,49^ so it is important to assess the impact of these processing variables on auxeticity. Building on these considerations, we evaluate the mechanical properties of CNF films with varied thicknesses and densities. Figure 3a shows the mechanical properties of CNF films made from different CNF suspension amounts. For this study, the aspect ratio is maintained at 5 because this aspect ratio gave the largest auxetic response for the CNF-270 films, as previously discussed. Here, the elastic moduli of the films varied from 5 to 8 GPa and tensile strengths varied from 72 to 115 MPa. These values resemble those found in other studies.^50−52^ The elastic modulus of CNF-90 films is statistically different compared with those of the other films; however, the other films have similar elastic modulus values. This result can be attributed to the higher porosity of the CNF-90 films. Previously, Henriksson et al. found that increasing porosity from 19% to 40% in CNF papers resulted in a 49.7% decrease in the elastic modulus and a 53.7% decrease in tensile strength.^44^ To determine whether the lower elastic modulus in CNF-90 is due to the porosity and the resulting level of fiber–fiber interactions rather than fiber binding strength, the elastic modulus is normalized by the density of films in Table S3. In cellulose fiber films, binding strength refers to the degree of adhesion between individual fibers within the film network.^53^ Normalizing elastic modulus accounts for variation in density/porosity and isolates the contribution of fiber binding strength to mechanical properties.^54^ From Table S3, it is evident that CNF-90 and CNF-270 films have similar normalized elastic modulus values, indicating similar binding strength. Given that all CNF films were made by following the same procedure, the differences in mechanical behavior stem from variations in their 3D network structure and porosity distribution. Similarly, the tensile strength of CNF-90 films is much lower than the tensile strength of the CNF-360 and CNF-450 films. On the other hand, the CNF-270, CNF-360, and CNF-450 films show similar tensile strengths as they have similar porosities and densities as described in Table 1. Thus, this increase in tensile strength can be attributed to the increased density and lower porosity.

The thickness strain values from elastic deformation shown in Figure 3b further illustrate the effect of the physical structure of the film on the auxetic response of the CNF films. The CNF-90 and CNF-180 samples show a consistent decrease in thickness with extension, indicative of positive Poisson’s ratio values. This response is attributed to increased porosity in the films and the more open structure. On the contrary, CNF-270, CNF-360, and CNF-450 films show expansion in thickness initially with axial stretching. These three films show similar auxeticity in the elastic region, even though their thickness increases with increasing amount of suspension. This similar auxetic behavior among these films can be ascribed to their comparable density (or porosity). The thickness strain as a function of axial strain taken until tensile failure for these films is shown in Figure S7a. In the plastic region, the CNF-360 and CNF-450 films show a much lower auxetic response compared to the CNF-270 films, even though these films are made with the same nanofibrils and fabrication process. Further analysis of the structural differences in these films that lead to the different auxetic response in the plastic region is presented later, as it is important to consider how the film microstructure can impact auxeticity. Specifically, the fiber arrangement and porosity distribution have been shown to play a significant role in the auxeticity of fiber networks.

The instantaneous Poisson’s ratios calculated for the CNF films with various thicknesses and densities in the elastic region are plotted in Figure 3c. It is clear from the data that the CNF-90 and CNF-180 films do not show auxeticity in the elastic region. On the contrary, CNF-270, CNF-360, and CNF-450 films show negative instantaneous Poisson’s ratios during elastic deformation. The Poisson’s ratios of the same samples calculated using linear fitting, instantaneous, and effective strain are shown in Table 3. CNF-90 and CNF-180 films show a decreasing effective (Figure S8a) and instantaneous (Figure S7b) Poisson’s ratio with increasing axial strain; however, the values remain positive until tensile failure. These results indicate that the CNF-90 and CNF-180 films do not show auxetic behavior. The Poisson’s ratios of CNF-270, CNF-360, and CNF-450 films are all negative values in the elastic region and thus have an auxetic response. The largest instantaneous Poisson’ ratio value of −7.99 is exhibited by the CNF-270 film at 0.4% strain. The CNF-360 samples start with an instantaneous Poisson’s ratio of −1.83 at 0.2% strain, which increases in magnitude to −2.62 at 0.4% strain and then continues to decrease in the elastic region. CNF-450 samples exhibit the largest negative instantaneous Poisson’s ratio of −5.38 at 0.4% strain in the nonplastic region. The linear fitting Poisson’s ratio values for CNF-270, CNF-360, and CNF-450 films are −5.3, −1.83, and −3.97, respectively (Figure S8b). The auxetic behavior in the elastic region of these CNF films having 1 g/cm^3^ density is similar to those reported for cellulose-based paper and paperboards. For example, copy paper and paperboard show Poisson’s ratios of −3 and −1.1, respectively, when calculated by linear fitting of the elastic region (up to 0.7% strain).^17^ Stenberg et al. have reported negative out-of-plane Poisson’s ratios of −0.5 to −4.5 in various sack papers, newspapers and coated/uncoated paperboards both in machine and nonmachine direction.^28^ Previous investigations by Öhrn, Baumgarten and Göttsching, and Mann et al. have reported that the out-of-plane Poisson’s ratios in various kraft papers and hand sheets can range from 1 to −5.^29,55,56^Table S2 compares the Poisson’s ratios (calculated by linear fitting in the elastic region) of previously reported values of various papers from Verma et al. with the current study.^17^ This similarity is in line with our hypothesis that CNF films would exhibit an auxetic response similar to that of cellulose-based papers, as their network structures are similar.

**Table 3:** Poisson’s Ratios for CNF Films in Varying Suspension Amounts Calculated in the Elastic Region

		**instantaneous Poisson’s ratio**				**effective Poisson’s ratio**			
**CNF films**	**linear fitting**	at 0.2% strain	at 0.4% strain	at 0.6% strain	at 0.8% strain	at 0.2% strain	at 0.4% strain	at 0.6% strain	at 0.8% strain
CNF-90	4.34	7.82 ± 7.15	2.49 ± 11.07	2.64 ± 5.91	2.1 ± 1.21	5.26 ± 7.21	3.91 ± 3.57	3.49 ± 1.95	3.27 ± 2.33
CNF-180	1.25	0.00 ± 0.00	4.19 ± 3.78	0.28 ± 4.89	–0.3 ± 7.00	0.00 ± 0.00	2.05 ± 1.89	1.45 ± 2.21	1.03 ± 1.98
CNF-270	–5.3	–6.15 ± 1.94	**-7.99 ± 5.49**	–4.29 ± 6.54	1.03 ± 5.52	–6.15 ± 1.94	**-7.12 ± 3.44**	–6.19 ± 1.93	–4.38 ± 2.38
CNF-360	–1.83	–2.62 ± 3.89	–1.74 ± 2.39	–0.85 ± 3.63	–1.77 ± 4.97	–2.62 ± 3.89	–2.18 ± 2.18	–1.74 ± 2.38	–1.74 ± 1.24
CNF-450	–3.97	–5.38 ± 1.88	–3.37 ± 4.15	–2.64 ± 4.36	–3.99 ± 3.74	–5.38 ± 1.88	–4.39 ± 2.64	–3.82 ± 2.38	–3.87 ± 1.03

The instantaneous Poisson’s ratio as a function of axial strain until tensile failure of CNF films made from different suspensions is shown in Figure S7b. In the plastic region, the thickness strain of CNF-270 films decreases slightly compared with the elastic region and remains stable until failure. On the contrary, CNF-360 and CNF-450 show much lower auxetic response compared to the CNF-270 films in the plastic region. As indicated by the instantaneous Poisson’s ratio in the plastic region, the films exhibit a near zero value of the instantaneous Poisson’s ratio. This result indicates that the thickness is not changing significantly at these levels of axial strain, and changes are occurring in the network structure.

### 3.3 The Mechanism of Auxetic Behavior in CNF Films

**Figure 4:**
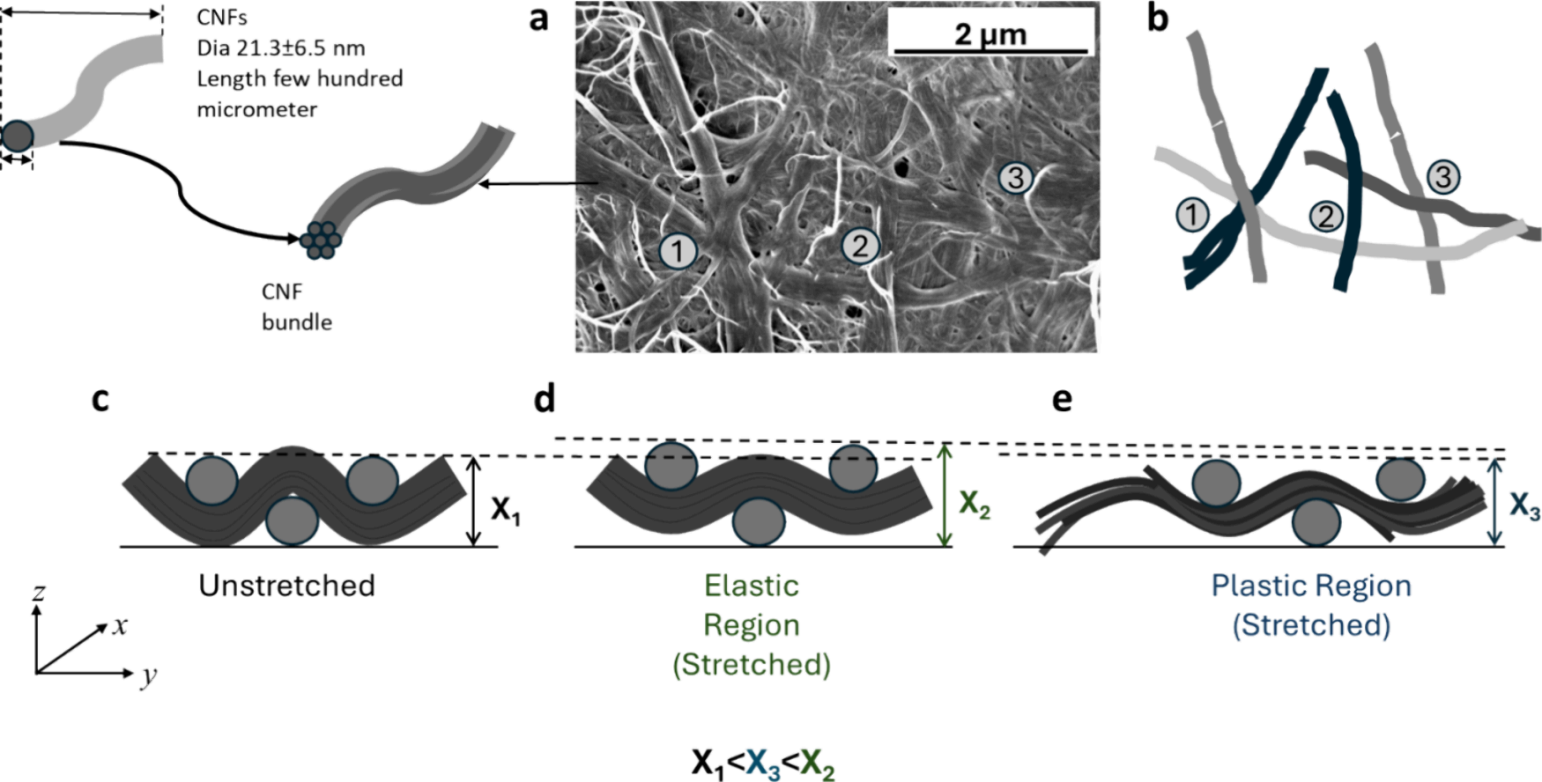
Mechanism of auxetic behavior in CNF films. (a) SEM morphology of the CNF film, positions 1–3 highlighting the CNF bundle junctions, (b) simplified depiction of the CNF bundle network that plays a key role in the auxetic response, (c) cross section of the three layers of fibers in unstretched and stretched conditions in (d) elastic and (e) plastic regions. (Parts c and d were adapted with permission from Verma, P.; Shofner, M. L.; Griffin, A. C., Deconstructing the auxetic behavior of paper. physica status solidi (b) 2014, 251 (2), 289–296. Copyright 2014 John Wiley and Sons).

Given the importance of the fiber network in the auxeticity and overall mechanical response of CNF films, understanding the mechanisms of auxetic behavior requires investigating fiber network formation and their overall microstructure. Cellulose fiber arrangement is inferred from high-resolution SEM micrographs of the top of the CNF film, shown in (Figure 4a). From the SEM micrographs, it is clear that multiple CNF nanofibrils agglomerate into CNF bundles, which are much thicker than individual nanofibrils. During the vacuum filtration of CNF suspensions, the water gradually passes through the filter paper, leaving the wet CNF film on top. During the first stage of dewatering, some agglomerates form CNF bundles that have much larger diameters than that of a single cellulose nanofibril, particularly near the filter film interface. The average diameter measured here varies from ∼20 to 40 nm and 1–2 μm. Also, the length of these bundles can extend as much as a few hundred micrometers to millimeters. As the dewatering stage advances, these CNF bundles form a random interwoven network, where one CNF bundle is attached to multiple other bundles along its length. The CNF bundles can interact or attach to multiple other bundles as shown in Figure 4a,b in positions 1, 2, and 3. These junctions of CNF bundles remain attached by interfiber hydrogen bonds, strengthening further during drying. Because of the random layout, these CNF bundles are not completely extended in the final dry films. Thus, during tensile loading of the CNF films, the flexible and relaxed bundles can pull at the contact points, increasing in thickness without having a sliding effect.

**Figure 5:**
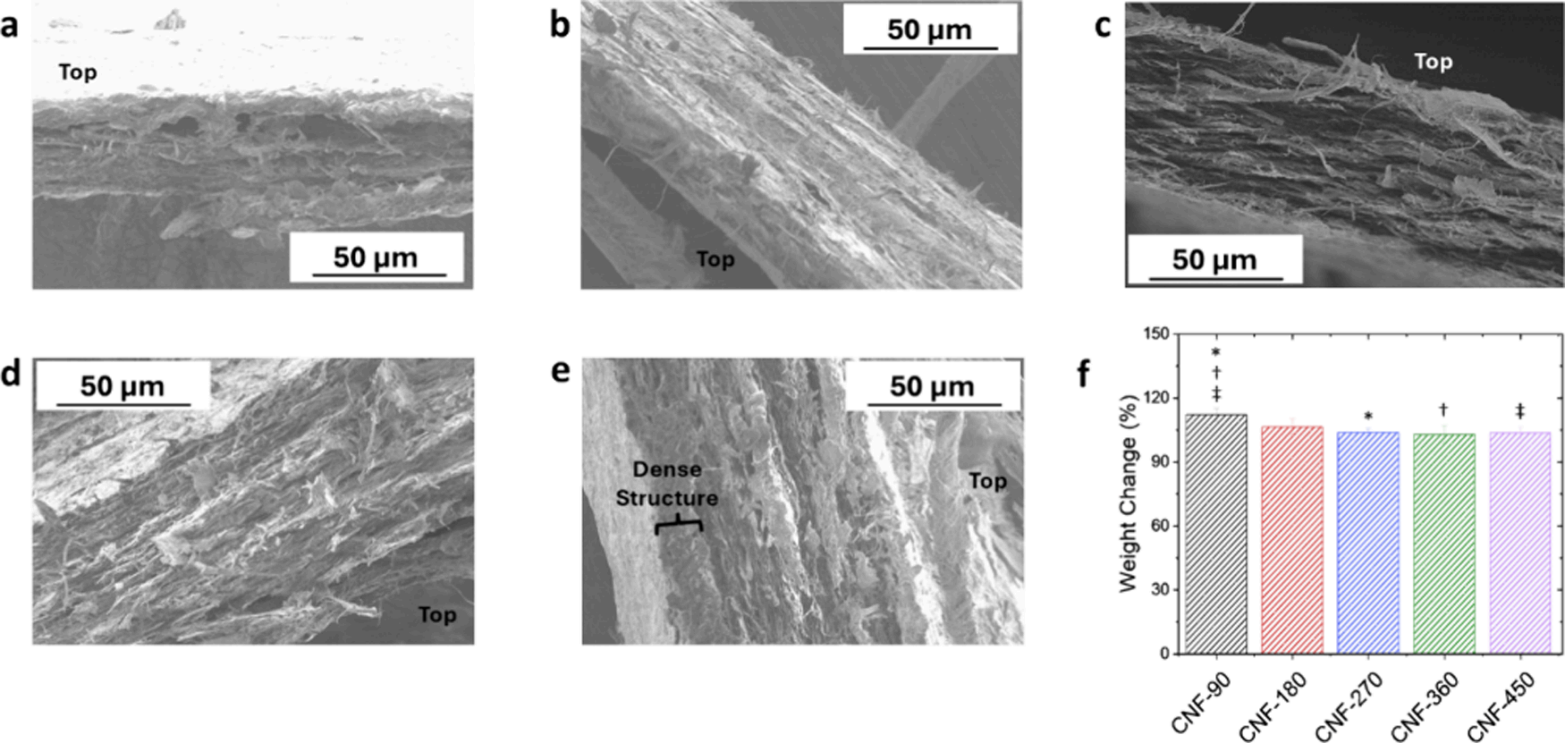
Cross-sectional morphology of (a) CNF-90, (b) CNF-180, (c) CNF-270, (d) CNF-360, and (e) CNF-450 films after tensile failure and (f) weight change after the swelling test. Values marked in the swelling test results with the same symbols are statistically different from each other.

To visualize the auxetic behavior, we present a simplified schematic showing a film cross section in Figure 4c, illustrating three layers of CNF bundles. In the unstretched condition, the fibers lie on top of each other with a thickness of X_1_. When the film is extended in the elastic region, the CNF bundle in the middle stretches and pushes away the fiber above and below it, leading to an increased thickness of X_2_. This effect becomes amplified in the CNF films as they consist of thousands of layered lamellar fiber structures, which is evident from the cross-sectional morphology shown in the SEM micrographs in Figure 5a–e. As a result, the overall thickness of the CNF film increases, resulting in out-of-plane auxetic behavior.

Our results from varying the aspect ratio of CNF samples have indicated that an external constraint is an important parameter in terms of demonstrating auxeticity in cellulose films. In the lower AR samples, the fiber network is constrained along the sample length by the grips. Thus, the extension of individual CNF bundles may not contribute to the overall auxetic response in shorter samples. Contrarily, samples with an aspect ratio equal to or greater than 5 have areas of the sample far enough away from the grips that they are not constrained by the grips. Since interactions between neighboring fibers are not constrained by external factors, these junctions can induce a continuous reorganization of the nanofibers during tensile loading, thus exhibiting a more pronounced auxetic response in longer samples.

The auxetic behavior in the elastic region of CNF films is analogous to the auxetic behavior of cellulose-based copy papers.^17^ According to Verma et al.,^17^ copy paper fails at 1% strain in the elastic region with negligible plastic deformation. However, our results have revealed that in the plastic region, the auxetic response decreases compared to the elastic region in all sets of CNF films. It has been reported that the deformation in the plastic region of the cellulose films stems from bond breaking and fiber pull-out in the fibrous network.^57^ Henriksson et al. have reasoned that the plastic deformation is related to debonding and slippage of nanofibrils facilitated by porosity.^44^ Zhu et al. have suggested that in the inelastic region of the stress–strain curve, interfiber slippage can cause breaking and reorganization of hydrogen bonds, which results in the high toughness of the cellulose nanopapers.^58^ Mao et al. have proposed that the plastic deformation is associated with breaking of hydrogen bonds within the disordered regions of cellulose nanofibers.^59^ From these studies, it is inferred that the reason for the reduction of the auxetic response in the plastic region is due to the breakdown of hydrogen bonds between cellulose nanofibers and interfiber slippage. A visualization of this behavior is shown in Figure 4e, which results in a thickness denoted as X_3_. This behavior amplifies in the layered fiber structure, and thus, the auxetic response keeps reducing in the plastic region until failure occurs in CNF-360 and CNF-450 films. However, overall, the network remains largely intact until failure, and only small changes are seen in the thickness strain values associated with the plastic axial strain for CNF-270 films.

Cross-sectional SEM images of all of the CNF samples after failure show a lamellar layered structure (Figure 5a–e). The CNF-90 film displays a more porous and open structure (Figure 5a), particularly near the top of the film that was not in contact with the filter paper when it was fabricated. It can also be seen that the CNF-270 film has the most uniform layered structure compared to the other films (Figure 5c). On the other hand, the CNF-450 film shows a graded difference in the structure across the thickness with a denser structure at the bottom compared to the top layers of the film (Figure 5e). The formation of nonuniform layers stems from the variable vacuum strength between the first stage of dewatering through the end of filtration, initially the vacuum pressure is stronger and as dewatering progresses the formation of fiber layers lower the vacuum pressure at the top. As a result, as the film forms from bottom to top, the film layers change from dense layers to more open layers, showing larger porosities. This gradient in porosity can lead to weaker mechanical properties.^60,61^ In our case, these porosities lead to lower levels of fiber–fiber interaction and thus lower auxetic response. This nonuniform distribution of porosities is one of the reasons why the CNF-360 and CNF-450 films had a similar auxetic response to the CNF-270 film in the elastic region, even though they have comparative density and higher thickness. Correspondingly, these nonuniform porous layers contribute to the lower auxetic response of the CNF-360 and CNF-450 films in the plastic region.

Given that fiber–fiber interactions play a significant role in the auxeticity of fiber networks,^62−64^ swelling tests are performed to further estimate the porosity and fiber–fiber interaction in these CNF films. In this test, water fills the void spaces of the films, and as the saturation continues, the fiber network swells as it absorbs more water.^65,66^ This uptake of water causes an expansion in the overall volume and weight of the material. It can be further inferred that the amount of porosity and level of fiber–fiber interactions are inversely related.^44^ The weight change values are listed in Figure 5f. The data showed that the CNF-90 film has the highest porosity that is statistically different from CNF films made from higher suspensions and, as a result, the lowest expected level of fiber–fiber interaction among these films. Following this logic for the CNF-180, CNF-270, CNF-360, and CNF-450 films, the degree of fiber–fiber interactions is similar even though the CNF-180 film had a lower density than the other three types of films. On this basis, the 3D microstructure plays a bigger role in the auxetic response compared to the density or thickness of the films.

## 4 Conclusions

In this study, it is demonstrated that CNF films with a density of 1.05 g/cm^3^ and an aspect ratio of 5 show the largest negative Poisson’s ratio of −5.3 (for linear fitting) in the elastic region and the largest instantaneous Poisson’ ratio value of −7.99 at 0.4% strain. Varying the CNF suspension affected the film density, thickness, and weight. CNF-270, CNF-360, and CNF-450 films exhibit similar auxetic response with axial stretching in the elastic region, despite their thickness increasing with an increasing amount of suspension. The linear fitting in the elastic region reveals that for CNF-270, CNF-360, and CNF-450 films, Poisson’s ratio values are −5.3, −1.83, and −3.97, respectively. The auxetic response is attributed to the levels of fiber–fiber interaction between the cellulose nanofibril bundles at contact points. The difference in the values of Poisson’s ratio for different types of films is due to the film fabrication method influencing the fiber–network microstructure and distribution of porosity. Taken together, the results obtained in this study provide a framework for understanding the relationship between film processing and the resulting auxetic response, which has not yet been established. With this knowledge, further research can be conducted to capitalize on the auxeticity of CNFs in a variety of device designs.

## Supporting Information Available

Thermogravimetric analysis of CNF films, modified stress–strain curve compared to the general tensile test, effect of strain rates on auxetic response, thickness strain and Poisson’s ratio as a function of axial strain for CNF films with varying aspect ratios, thickness and density (PDF)
