# Supplementary material for: Out-of-Plane Auxetic Behavior in Cellulose Nanofibril Films
Source: ACS Omega. 2025 Mar 26;10(13):13339–49. doi: 10.1021/acsomega.4c09915 (PMC11983352; doi:10.1021/acsomega.4c09915)
Supplement: Supplementary file 1 — ao4c09915_si_001.pdf [file ao4c09915_si_001.pdf]

Supplementary Information

Out-of-Plane Auxetic Behavior in Cellulose Nanofibril Films

Fariha Rubaiya[1,2], Meisha L. Shofner[1,2*], Lauren M. Garten[1,2*]

[1] School of Materials Science and Engineering, Georgia Institute of Technology, Atlanta, GA 30332

[2] Renewable Bioproducts Institute, Georgia Institute of Technology, Atlanta, GA 30332

*Corresponding author's email: meisha.shofner@mse.gatech.edu & lauren.garten@mse.gatech.edu

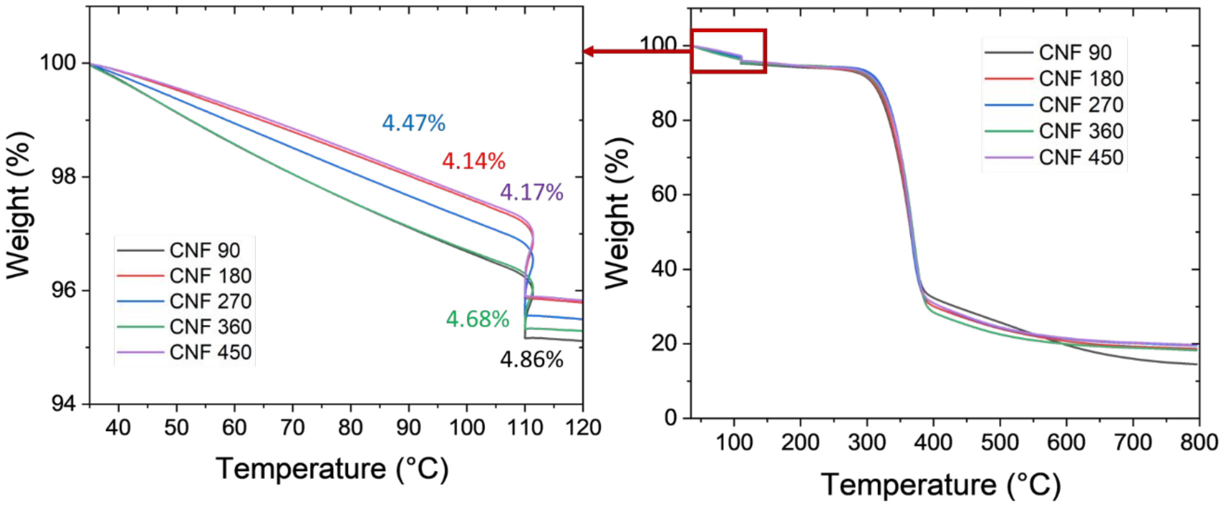

Figure S1. Weight loss data from TGA experiments of CNF films

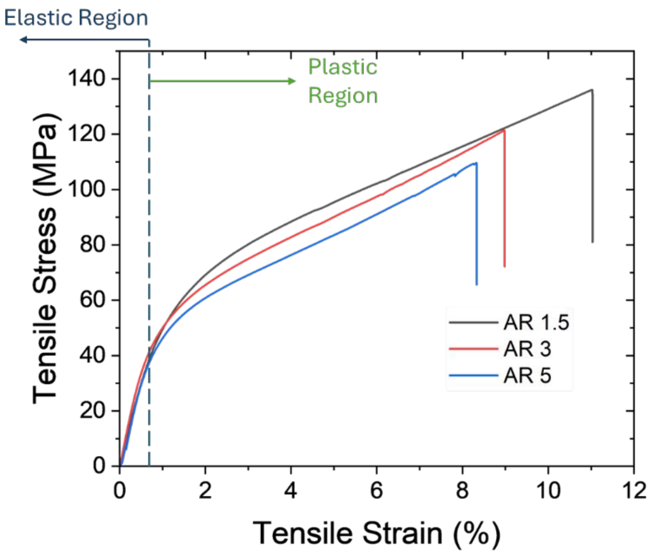

Figure S2. Stress-strain curve of the continuous tensile test of CNF-270 films with varying aspect ratios. (only the curve corresponding to the sample with maximum tensile strength is presented)

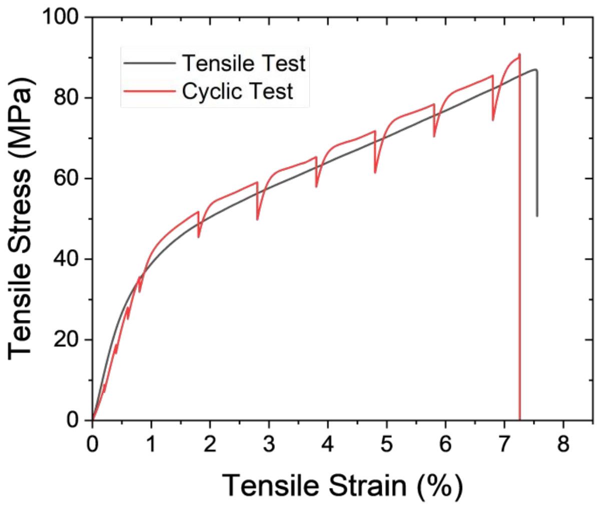

Figure S3. Stress-strain curve of the continuous tensile test overlapping the modified tensile test with intervals. The drop in stress when the test is paused is indicative of stress relaxation.

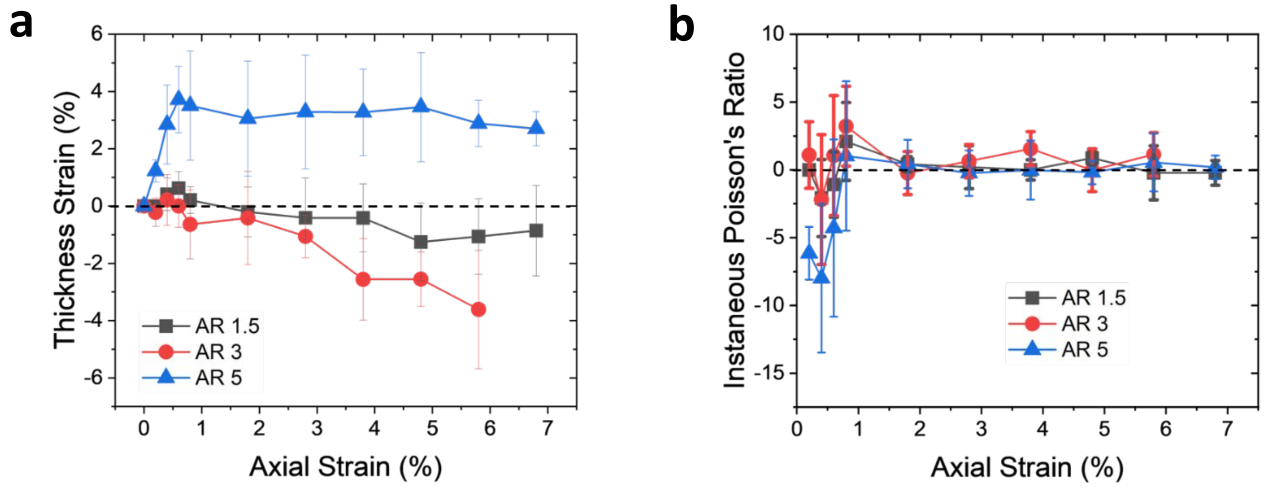

Figure S4. (a) Thickness strain and (b) instantaneous Poisson's ratio as a function of axial strain of CNF-270 films at various sample aspect ratios.

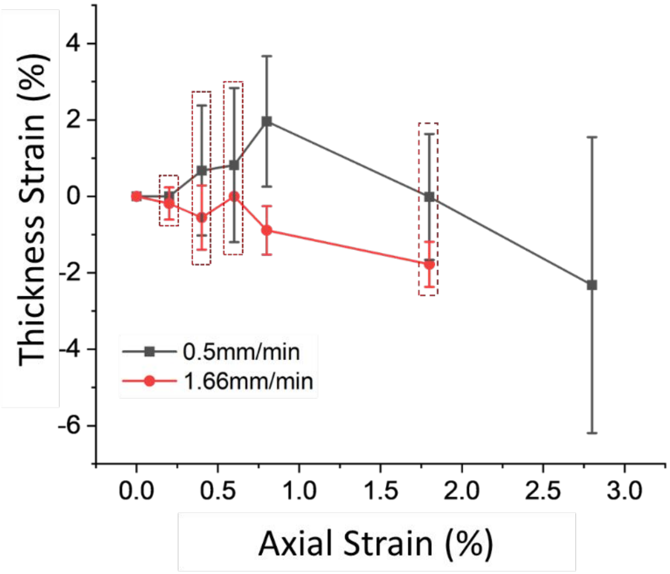

Figure S5. Thickness strain as a function of axial strain of CNF-270 films at various strain rates.

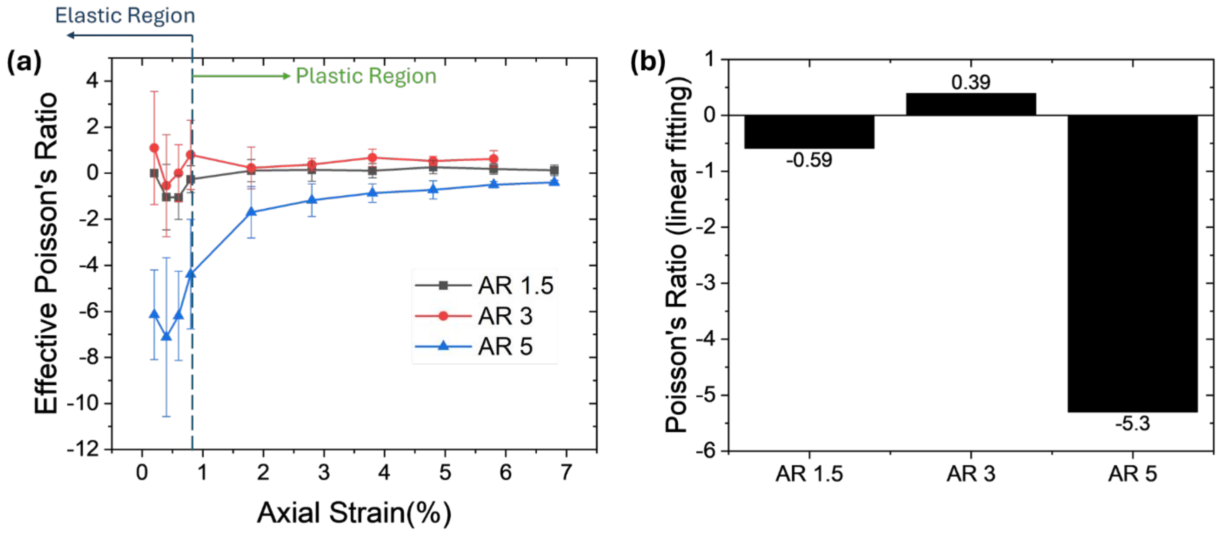

Figure S6. (a) Effective Poisson's ratio as a function of axial strain and (b) Poisson's ratio calculated via linear fitting (slope of the thickness strain vs axial strain) over the elastic regions of CNF-270 films at various sample aspect ratios.

Table S1. Average values of instantaneous Poisson's ratio for CNF-270 films until tensile failure

Strain (%)	Instantaneous Poisson's Ratio		
	AR 1.5	AR 3	AR 5
0.2	0.00	1.10	-6.15
0.4	2.84	-2.19	-7.99
0.6	2.41	1.06	-4.29
0.8	2.87	3.22	1.03
1.8	0.57	-0.23	0.43
`2.8	1.59	0.64	-0.24
3.8	0.75	1.56	-0.01
4.8	0.49	-0.02	-0.18
5.8	1.99	1.14	0.55
6.8	0.92		0.19

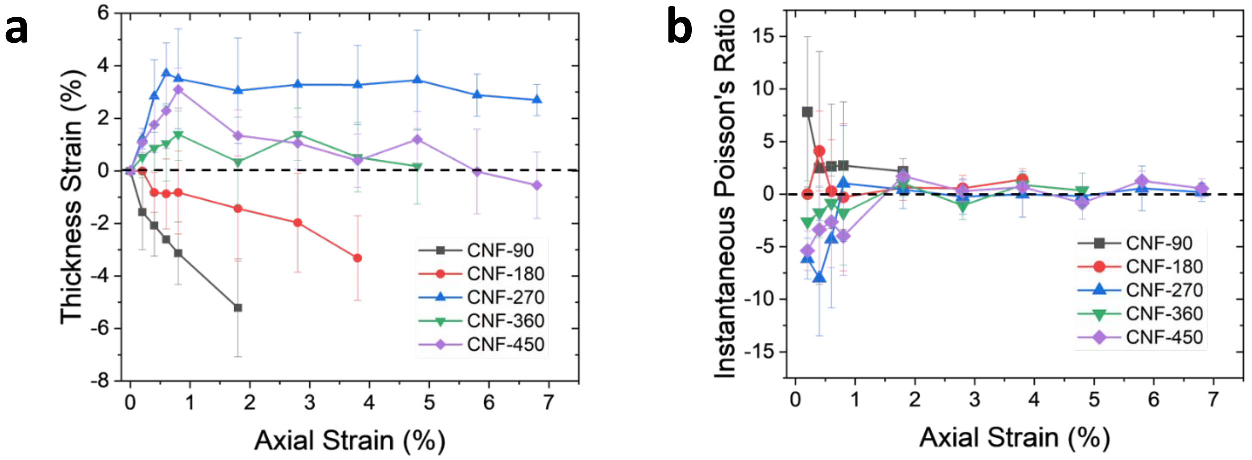

Figure S7. (a) Thickness strain and (b) Instantaneous Poisson's ratio as a function of axial strain of CNF films at various thickness and density.

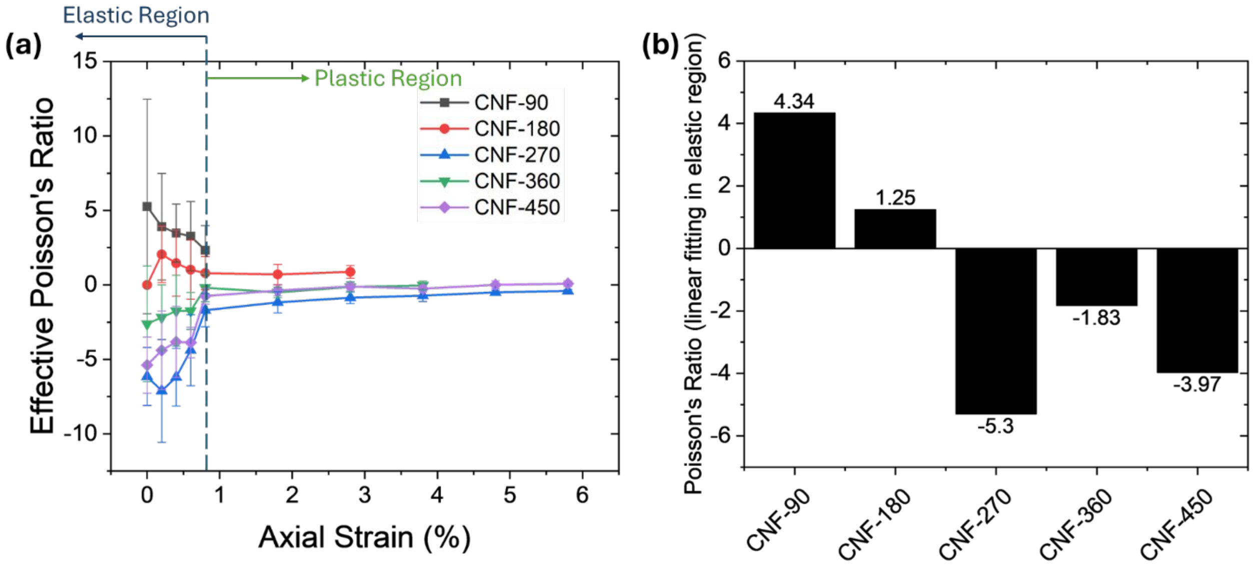

Figure S8. (a) Effective Poisson's ratio as a function of axial strain and (b) Poisson's ratio calculated via linear fitting (slope of the thickness strain vs axial strain) over the elastic regions of CNF films at various thickness and density.

Table S2. Comparison of Poisson's ratios (linear fit of elastic region) of CNF films made from various suspension of the current study with various papers from literature.

Sample	Thickness (μm)	Grammage (g/m^2)	Poisson' s Ratio (linear fit of elastic region)	References
copy paper	105 ± 2	75	-3.3	[1]
paperboard	270 ± 2	220	-1.1	[1]
bamboo paper	433 ± 7	295	0.1	[1]
cotton paper	190 ± 3	120	-0.3	[1]
filter paper	178 ± 4	85	-0.3	[1]
glassine paper	52 ± 2	48	1.2	[1]
CNF-90	44±3	34	4.3	Current Study
CNF-180	76±5	72	1.3	"
CNF-270	99±4	103	-5.3	"
CNF-360	130±8	135	-1.8	"
CNF-450	162±3	175	-3.9	"

Table S3. Comparison of normalized elastic modulus of CNF films with various thickness and densities suggesting similar binding strength across the films.

Sample	Thickness(μm)	Density (g/cm^3)	Elastic Modulus (GPa)	Normalized Elastic Modulus (elastic modulus/density)
CNF-90	44 ± 3	0.76 ± 0.03	5.32 ± 0.05	7.04 ± 0.45
CNF-180	98 ± 3	1.05 ± 0.04	6.78 ± 0.19	7.14 ± 0.12
CNF-270	98 ± 4	1.05 ± 0.03	6.72 ± 0.79	7.13 ± 0.48
CNF-360	130 ± 8	1.04 ± 0.03	6.92 ± 0.33	6.68 ± 0.27
CNF-450	162 ± 3	1.07 ± 0.02	7.28 ± 0.61	6.76 ± 0.43

References

1. Verma, P., M.L. Shofner, and A.C. Griffin, *Deconstructing the auxetic behavior of paper.* physica status solidi (b), 2014. **251**(2): p. 289-296.
